# Community-associated methicillin-resistant *Staphylococcus aureus* in the Kimberley region of Western Australia, epidemiology and burden on hospitals

**DOI:** 10.1017/S0950268824001201

**Published:** 2024-11-27

**Authors:** Lauren Edna Bloomfield, Geoffrey Coombs, Paul Armstrong

**Affiliations:** 1 Communicable Disease Control Directorate, WA Department of Health, Perth, WA, Australia; 2 School of Medicine, The University of Notre Dame, Fremantle, WA, Australia; 3 PathWest Laboratory Medicine, Fiona Stanley Hospital, Perth, WA, Australia; 4 School of Medical, Molecular and Forensic Sciences, Murdoch University, Perth, WA, Australia

**Keywords:** infectious disease epidemiology, Staphylococcus aureus, surveillance, burden of disease, community-associated infection, Australia

## Abstract

This study presents surveillance data from 1 July 2003 to 30 June 2023 for community-associated methicillin-resistant *Staphylococcus aureus* (CA-MRSA) notified in the Kimberley region of Western Australia (WA) and describes the region’s changing CA-MRSA epidemiology over this period. A subset of CA-MRSA notifications from 1 July 2003 to 30 June 2015 were linked to inpatient and emergency department records. Episodes of care (EOC) during which a positive CA-MRSA specimen was collected within the first 48 hours of admission and emergency presentations (EP) during which a positive CA-MRSA specimen was collected on the same day as presentation were selected and analysed further. Notification rates of CA-MRSA in the Kimberley region of WA increased from 250 cases per 100,000 populations in 2003/2004 to 3,625 cases per 100,000 in 2022/2023, peaking at 6,255 cases per 100,000 in 2016/2017. Since 2010, there has been an increase in notifications of Panton-Valentine leucocidin positive (PVL^+^) CA-MRSA, predominantly due to the ‘Queensland Clone’. PVL^+^ CA-MRSA infections disproportionately affect younger, Aboriginal people and are associated with an increasing burden on hospital services, particularly emergency departments. It is unclear from this study if PVL^+^ MRSA are associated with more severe skin and soft-tissue infections, and further investigation is needed.

## Introduction

*Staphylococcus aureus* is one of the most frequently isolated bacterial pathogens in humans and is an important cause of skin and soft-tissue infections (SSTIs), pneumonia, septic arthritis, endocarditis, osteomyelitis, foreign-body infections, and sepsis [1]. Historically, methicillin-resistant *S. aureus* (MRSA) was primarily confined to healthcare environments and was referred to as healthcare-associated MRSA (HA-MRSA) [2]. However, over the last 30 years there has been an increase in the number of MRSA infections in persons without the usual healthcare-associated risk factors and an increased recognition of new MRSA clones, collectively known as community-associated MRSA (CA-MRSA) [1]. The increasing incidence of CA-MRSA has been reported globally [3, 4] and has been associated with an increased burden on hospitals via dissemination and outbreaks within healthcare facilities. [5]

In Australia, CA-MRSA infections first emerged in Western Australia (WA) in the early 1990s [6]. Since this time, several studies have noted a heavy burden of staphylococcal disease and an increasing prevalence of CA-MRSA in Aboriginal people across northern Australia [2, 7]. Furthermore, Aboriginal people infected with CA-MRSA are more likely to develop severe clinical manifestations compared to non-Aboriginal people [8, 9]. Facilitated by local and international travel, a number of CA-MRSA clones circulate throughout Australia [10].

Since 2008, the most frequently isolated CA-MRSA clone in Australia is ST93-IV, colloquially known as ‘Queensland CA-MRSA’ or the ‘Queensland clone’. ST93-IV contributes to the increasing proportion of CA-MRSA in Australia that contain the Panton-Valentine leucocidin (PVL)-associated genes [11]. Evidence suggests ST93-IV emerged from north-western Australia before proliferating across the country. The ‘IV’ refers to the SCC*mec* type, the mobile element that harbours the *mecA* gene that codes for the PBP2a protein associated with the MRSA phenotype [12]. The PVL protein is an exotoxins associated with enhanced pathogenicity [1, 13]. PVL-positive (PVL^+^) *S. aureus* is highly transmissible and may cause recurrent SSTIs despite antibiotic treatment [14–16].

In WA, MRSA is a notifiable organism, and surveillance has shown notification rates of CA-MRSA in the Kimberley region have increased more rapidly than in any other health region in the state [6]. Given the rapid rate of increase of CA-MRSA in the Kimberley and the large proportion of residents in this region who are at increased risk of severe disease, this study aims to explore and quantify the burden CA-MRSA infections have on hospitals, inpatient facilities, and emergency departments. We examine changes in the CA-MRSA notification rate, clone type, and clone PVL status, as well as the number and characteristics of hospital admissions and emergency presentations associated with the isolation of CA-MRSA.

## Methods

### Data sources

Notification data for MRSA cases referred to the PathWest Gram-positive Typing Laboratory (GPTL) were linked to inpatient hospitalisation and emergency department (ED) data by the WA Health Data Linkage Branch using probabilistic linkage methods as described by Holman et al. [17]. Notifications were linked using a unique identifier to each hospitalisation encounter. Molecular testing methods have previously been described; briefly, the classification of CA-MRSA clones is determined by a combination of the molecular analysis of the seven housekeeping gene sequence types using MLST and the SCC*mec* type using multiplex PCR [10, 18].

There are six hospitals (all with emergency departments) in the Kimberley region, all of whom contribute data to the inpatient and ED data collections. All available inpatient and ED records were retrieved for a period of 6 months prior to the date of notification, up until the date of data extraction.

#### MRSA notifications

The following variables from this data set were used in the analysis: unique identifier for each notification, sex, collection date, MRSA clone type, and reason why the specimen was collected. Notification data were extracted with the following inclusion criteria:

- Collection date between 1 July 2003 and 30 June 2023.
- Persons with a Kimberley region address (classified by residential postcode) at the time a microbiology specimen was collected.
- The specimen was not identified as a duplicate (i.e. an isolate of the same MRSA clone collected from the same patient on the same day).
- The specimen was positive for a CA-MRSA clone, characterised by the GPTL [10, 18].
- The specimen was identified as a clinical specimen (i.e. screening specimens excluded).

#### Emergency department data

Emergency department data were obtained from the Western Australian Department of Health Emergency Department Data Collection (EDDC) [19] and provided by the Western Australian Department of Health Data Linkage Branch. The EDDC contains data on emergency department activity in WA’s public hospitals and in emergency department activity from private hospitals under contract with the Western Australian Government. EDDC Emergency Department data were obtained between 01 July 2003 and 30 June 2015 (herein referred to as ‘the study period’).

The following variables from this data set were used in the analysis: unique identifier for each presentation (linkable to the MRSA notifications), presentation date, major diagnostic category of the presentation, and discharge location (used to determine if a case was admitted).

#### Inpatient data

Inpatient data were obtained for the study period from the Western Australian Department of Health’s Hospital Morbidity Data Collection (HMDC) [20]. The data excluded boarders, healthy newborns, organ procurements, and aged care residents. Inpatient (day) admissions for extracorporeal dialysis, radiotherapy, and chemotherapy were also excluded. HMDC data includes public hospitals (acute and psychiatric) and private hospitals and day surgeries licenced by Western Australian Health.

The following variables from the data set were used in the analysis: unique identifier for each presentation (linkable to the MRSA notifications), admission date, separation (discharge) date, and ICD-10-AM codes for diagnoses and procedures. Episodes of care (EOC) where a positive CA-MRSA specimen was collected within 48 hours of initial admission were selected for further analysis. Total length of stay, principal diagnosis, and additional diagnosis codes were also reviewed. Duplicate specimens collected in the first 48 hours were not considered unique EOC.

#### Population count data

Estimated resident population (ERP) figures from 2003 to 2022 were obtained from the Australian Bureau of Statistics (ABS) [21]. The estimated resident population of the Kimberley grew from 33,026 in 2003 to 38,925 in 2022. Annual regional ERP were averaged across two calendar years to produce estimates for financial year periods. Aboriginal population estimates were derived by multiplying general population estimates by 41.6%, the stated proportion of Aboriginal and Torres Strait Islander people living in the Kimberley per ABS 2016 census data [22].

### Statistical analysis

Data analysis was performed using R version 4.2.1 [23]. Annual case counts were compared using Poisson regression, with the total regional population used as an offset. The Wilcoxon rank sum test was used to assess differences in median values of categorical variables. Chi-squared tests were used to assess significant differences for categorical data. Simple linear regression was used to compare differences in proportions between isolates and hospitalisations over time. A *p*-value of <0.05 was considered statistically significant.

### Ethics approval

Ethics approval was obtained from the Western Australian Department of Health Human Research Ethics Committee (Project 2014/47), the Western Australian Aboriginal Health Ethics Committee, and the Kimberley Aboriginal Health Planning Forum Research Subcommittee (Project number: RGS2816).

## Results

### Epidemiology of CA-MRSA in the Kimberley 2003–2023

**Figure 1.**
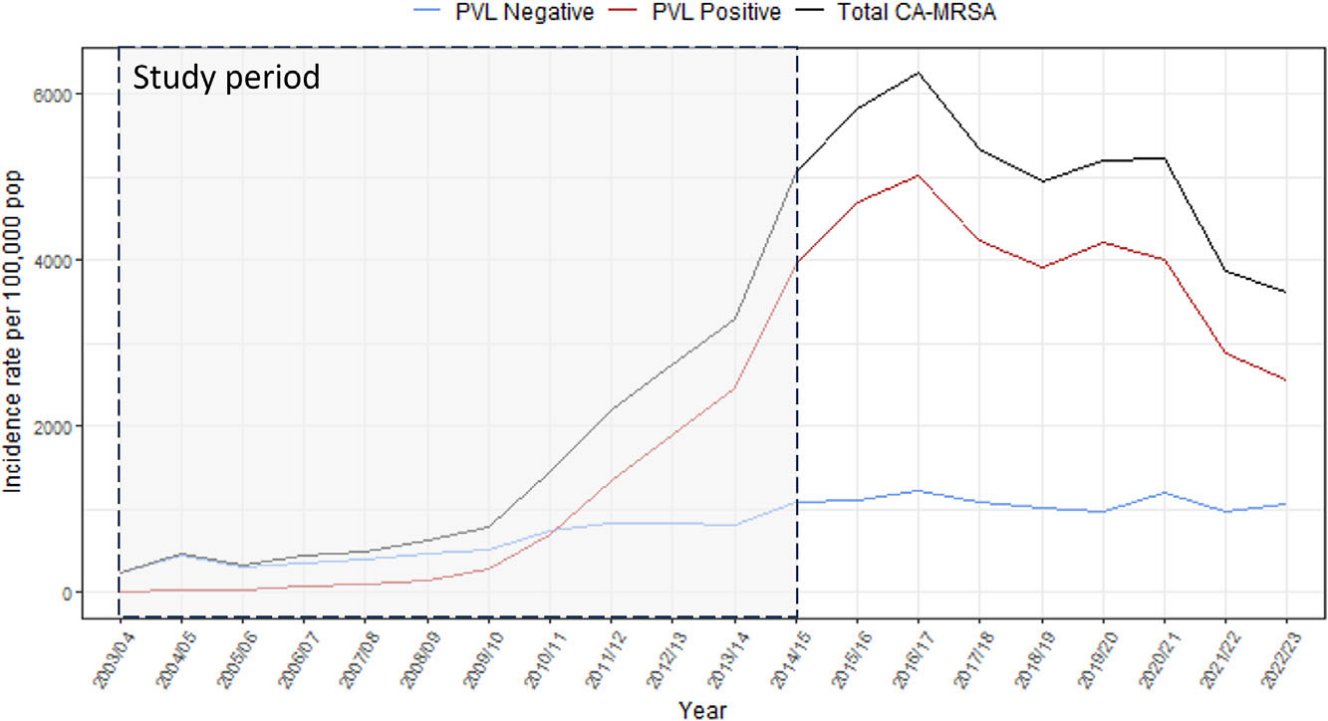
Annual CA-MRSA incidence rates, by PVL status, Kimberley region, 2003/2004–2022/2023.

Notification rates of CA-MRSA in the Kimberley region of WA increased from 250 cases per 100000 population in 2003/2004 to 3,625 cases per 100,000 in 2022/2023, peaking at 6,255 cases per 100,000 in 2016/2017. Annual incidence rates by PVL status are shown in Figure 1; rates increased year-on-year to a peak between 2014/2015 and 2016/2017, after which time they fluctuated before declining in 2020/2021.

#### Predominantly circulating clones

**Figure 2.**
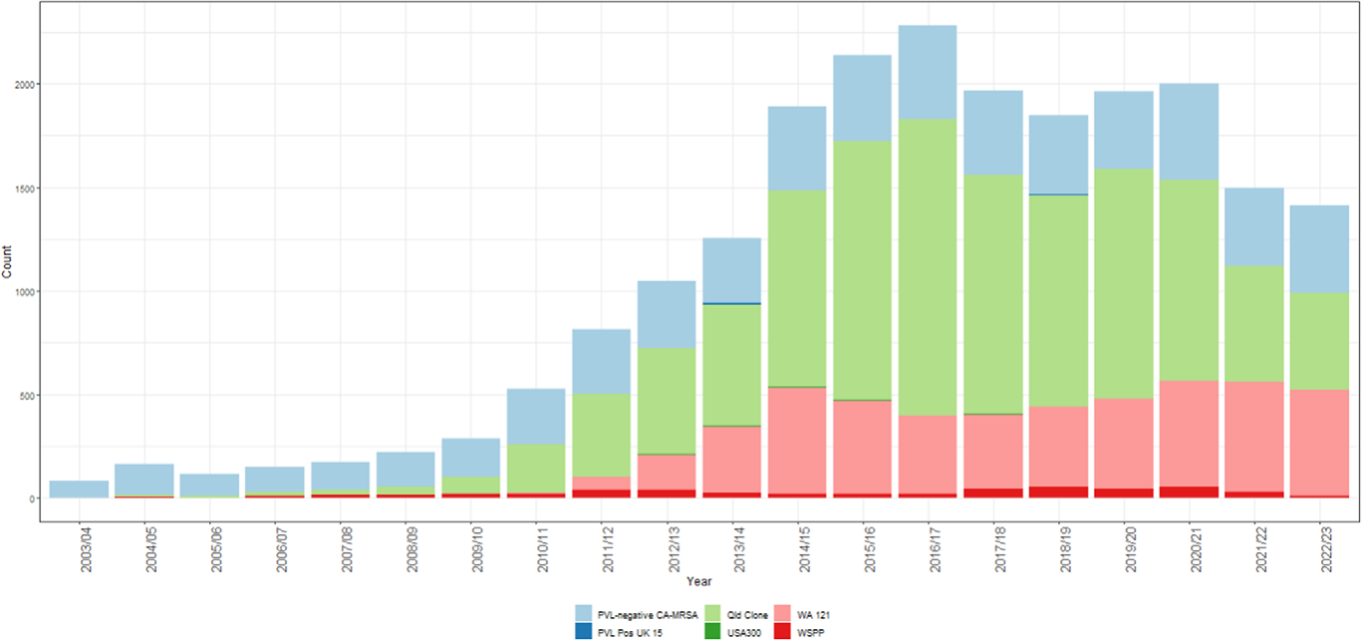
CA-MRSA strains notified in the Kimberley, 2003/2004–2022/2023.

The proportion of PVL^+^ CA-MRSA increased during the study period from 0% (*n* = 0) in 2003/2004 to 70% (*n* = 993) in 2022/2023 (chi-squared = 174.2, *p* < 0.001). PVL^+^ CA-MRSA accounted for 73.2% (*n* = 15,992) of all clinical MRSA isolates reported over the period. The majority (68%) of PVL^+^ CA-MRSA were identified as ST93-IV (“Queensland CA-MRSA”), with a further 30% identified as ST5-IV (colloquially known as “WA 121”). There was a notable shift in the pattern of PVL carriage commencing in 2009/2010 to predominantly PVL^+^ isolates, which has persisted. The recent decline in PVL^+^ rates observed between 2021/2021 and 2022/2023 has been driven by a decrease in the number of Queensland clone isolates, with relative stability in the number of WA 121 isolates reported since 2014/2015 (Figure 2).

### Case demographics – Hospital burden sub-study

For the hospital burden substudy using linked data, there were 6,117 unique CA-MRSA positive specimens that met the case definition, collected from 4,583 persons. Most cases contributed one specimen during the study period (median = 1, range 1–10).

Demographic data of cases reported during the study period, by PVL status, are shown in Table 1. A slightly larger proportion of specimens were from females (52.8%, *n* = 2,879 than males (47.1%, *n* = 3,229). Overall, 5344 (87.4%) CA-MRSA positive specimens were from Aboriginal persons, and 773 (12.6%) were from non-Aboriginal persons, demonstrating a significantly higher burden in this group than would be expected based on the reported proportion of Aboriginal residents living in this region (chi-squared = 3,434.1, *p* < 0.001).

**Table 1.** Demographics of MRSA cases in sub-study by PVL status, 2003/2004–2014/2015^a^

		PVL^−^ (*N* = 2,220)	PVL^+^ (*N* = 3,897)	Total (*N* = 6,117)
Age group	<10 years	461 (20.8%)	1,102 (28.3%)	1,563 (25.6%)
	11–19 years	183 (8.2%)	486 (12.5%)	669 (10.9%)
	20–19 years	314 (14.1%)	533 (13.7%)	847 (13.8%)
	30–39 years	369 (16.6%)	527 (13.5%)	896 (14.6%)
	40–49 years	325 (14.6%)	437 (11.2%)	762 (12.5%)
	50–59 years	258 (11.6%)	302 (7.7%)	560 (9.2%)
	60–69 years	127 (5.7%)	124 (3.2%)	251 (4.1%)
	70–79 years	63 (2.8%)	48 (1.2%)	111 (1.8%)
	≥80 years	42 (1.9%)	30 (0.8%)	72 (1.2%)
	Unspecified	78 (3.5%)	308 (7.9%)	386 (6.3%)
Gender	Female	1,173 (52.8%)	2,056 (52.8%)	3,229 (52.8%)
	Male	1,040 (46.8%)	1,839 (47.2%)	2,879 (47.1%)
	Other	7 (0.3%)	2 (0.1%)	9 (0.1%)
Aboriginal status	Aboriginal	1,903 (85.7%)	3,441 (88.3%)	5,344 (87.4%)
	Non-Aboriginal	317 (14.3%)	456 (11.7%)	773 (12.6%)

a Includes non-binary, unspecific and unknown.

The median age for Aboriginal CA-MRSA positive cases was significantly younger than non-Aboriginal cases (26 vs. 40, *p* < 0.001). Similarly, the median age for PVL^+^ cases was significantly younger than PVL-negative (PVL^−^) cases (24 vs. 33, *p* < 0.001).

### Burden on hospital services

#### Emergency departments

Notifications were linked to state-wide emergency department records. Emergency presentations (EP) where a positive CA-MRSA specimen was collected on the same day as presentation to the emergency department were selected for further analysis. Across the study period, there were a total of 2,265 EP, 1,683 of which were designated ‘CA-MRSA-related’ (presenting complaint under Major Diagnostic Category [MDC] 9 *Diseases and disorders of the skin, subcutaneous tissue and breast* or MDC 18 *Infectious and parasitic diseases*). Most cases had only one CA-MRSA-related EP during the study period (median = 1, range 1–6).

**Figure 3.**
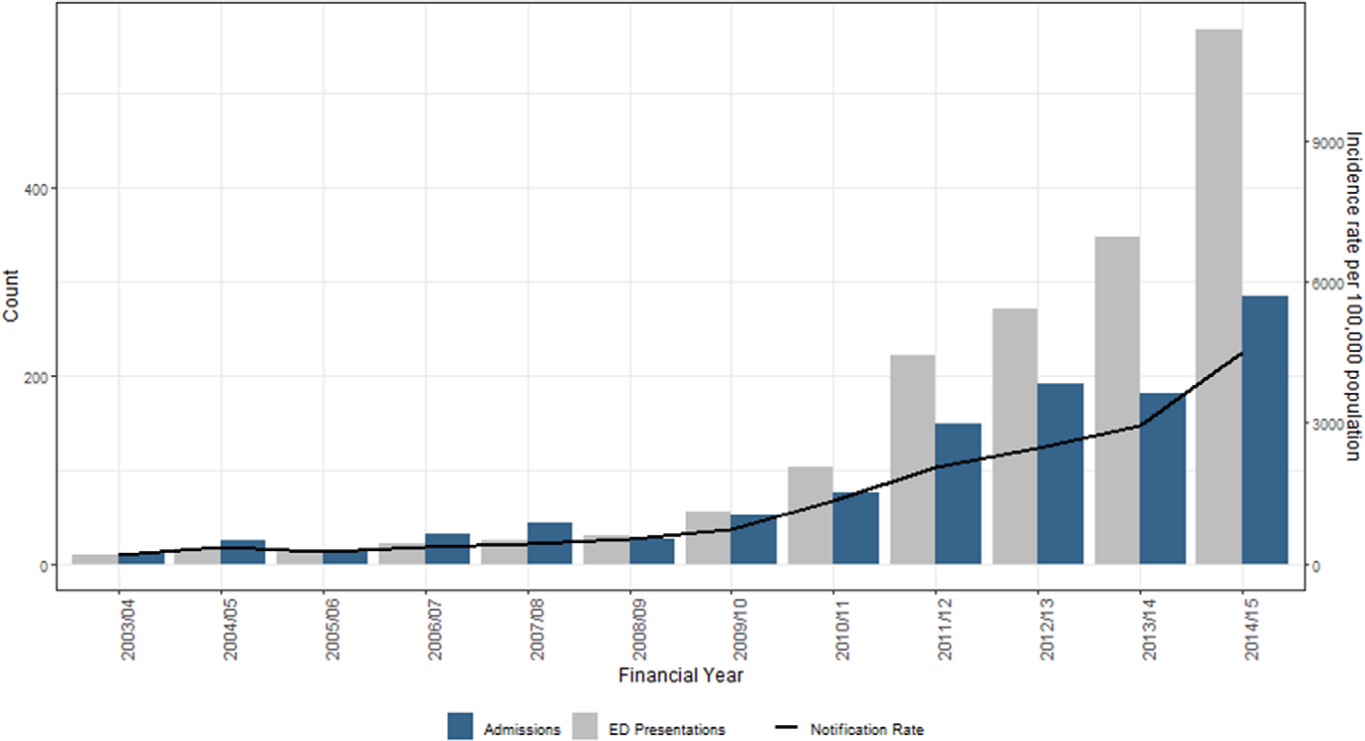
MRSA related ED presentations, admissions and notification rates, Kimberley, 2003/2004–2014/2015.

The total number of CA-MRSA-related EP increased each year in line with increasing case numbers (Figure 3). Using 2003/2004 presentations as baseline ‘expected levels’, an additional 1,553 additional ED presentations were reported over the study period due to the increased number of CA-MRSA cases.

#### Inpatient services

The 4,583 cases with at least one CA-MRSA notification over the study period were also linked to state-wide inpatient records. Across the study period, 1,251 CA-MRSA notifications from Kimberley residents were collected within 48 hours of admission to a hospital (considered ‘community-acquired’ rather than ‘hospital-acquired’). Most cases had one CA-MRSA-related EOC during the study period (median = 1, range 1–6). The ICD-10-AM principal diagnosis codes for the 1,251 EOC are shown in Table 2. The most frequent principal diagnosis group was *Chapter 12 Diseases of the skin and subcutaneous tissue*, with 44% of EOC having a diagnosis code in this chapter.

**Table 2.** Principal diagnosis groups for CA-MRSA cases identified within 48 hours of inpatient admission

Principal diagnosis group	PVL^−^	PVL^+^	Total
Diseases of the skin and subcutaneous tissue	117	356	473
Injury, poisoning and certain other consequences of external causes	109	104	213
Certain infectious and parasitic diseases	23	27	50
Diseases of the respiratory system	28	20	48
Diseases of the musculoskeletal system and connective tissue	14	21	35
Diseases of the genitourinary system	12	21	33
Endocrine, nutritional and metabolic diseases	18	12	30
Diseases of the circulatory system	15	7	22
Symptoms, signs and abnormal clinical and laboratory findings, not elsewhere classified	12	6	18
Diseases of the digestive system	8	9	17
Pregnancy, childbirth and the puerperium	9	8	17
Mental and behavioural disorders	7	3	10
Factors influencing health status and contact with health services	6	1	7
Diseases of the ear and mastoid process	2	4	6
Neoplasms	0	4	4
Diseases of the nervous system	2	2	4
Diseases of the blood and blood-forming organs and certain disorders involving the immune mechanism	0	3	3
Diseases of the eye and adnexa	0	3	3
Certain conditions originating in the perinatal period	2	1	3
Congenital malformations, deformations and chromosomal abnormalities	1	0	1

EOC were also further classified into those determined by clinician review as being likely to be CA-MRSA related, selecting only notifications with principal or additional diagnostic codes shown in Supplementary Material, Appendix 1. The ICD-10-AM codes reflect a range of SSTIs, as well as infective conditions such as endocarditis, *Staphylococcus*-related pneumonia, sepsis, and wound infections.

Overall, 1,084 (87%) of the EOC were designated as CA-MRSA-related, increasing across the study period in line with increasing CA-MRSA case notifications, from 11 in 2003/2004 to 285 in 2014/2015 (Figure 3). On average, 19% (range 13–28%) of all CA-MRSA notifications per year in the Kimberley were admitted to the hospital for an episode of care with a CA-MRSA-related diagnosis.

Using 2003/2004 admissions as baseline ‘expected levels’, an additional 941 admissions were reported over the study period due to the increasing number of CA-MRSA infections. This equates to an additional 2,823 bed days (assuming median length of stay [LOS] of 3 days) attributable to increasing case numbers.

### Differences in markers of severity by PVL status

**Figure 4.**
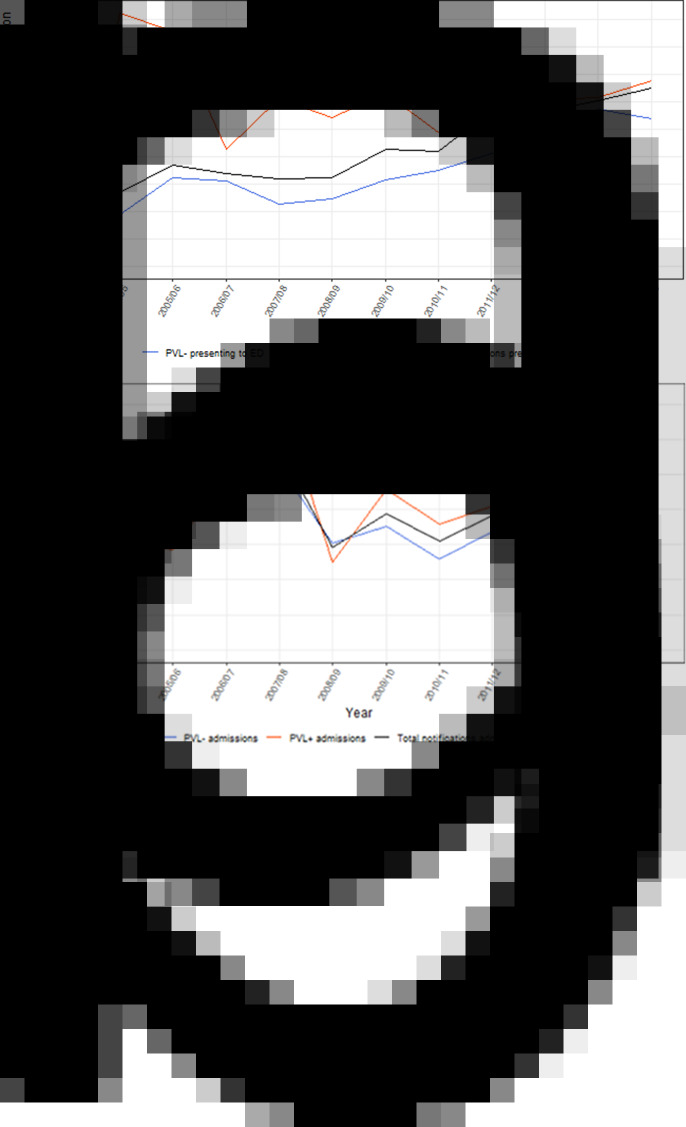
Proportion of total CA-MRSA notifications presenting to an ED with a CA-MRSA-related diagnosis (a) or admitted to hospital with a CA-MRSA-related diagnosis (b).

Although the data suggest PVL^+^ CA-MRSA may be causing more severe SSTIs, there was no evidence PVL^+^ CA-MRSA caused more severe systemic infections resulting in longer hospitalisation or a disproportionate increase in hospital burden. The proportion of all CA-MRSA notifications from the Kimberley region presenting to EDs annually with a CA-MRSA related diagnostic code was 21% (range 13–32%, Figure 4a), with an increasing annual trend over the reporting period when regressed against the proportion of PVL^+^ isolates (*R*^2^ = 0.88, *p* = <0.001).

No increasing trend was observed in the proportion of CA-MRSA notifications admitted to hospital each year, with 19% of notifications on average resulting in a CA-MRSA-related hospitalisation (range 15–28%, Figure 4b); regression against the proportion of PVL^+^ isolates confirmed no increase in the proportion of people with a CA-MRSA related admission, despite a marked increase in the proportion of PVL^+^ isolates (*R*^2^ = 0.08, *p* = 0.36).

Of the 1,084 hospital admissions for CA-MRSA-related EOC, 395 (36%) of the CA-MRSA associated with the admission were PVL^−^ and 689 (64%) were PVL^+^, reflecting the overall proportion of PVL^+^ isolates collected during the study period (36% PVL^−^, 64% PVL^+^). The median LOS for PVL^−^ and PVL^+^ EOC was 3 days (range 1–80 days and 1–107 days, IQR 2–5 days and 2–6 days, respectively).

Admissions for SSTIs (ICD-10-AM codes shown in Supplementary Material, Appendix 2) accounted for 80% of CA-MRSA related EOC (867/1084). Overall, 43% of CA-MRSA-related SSTI admissions required aspiration and/or excision, incision, or drainage (ICD-10-AM codes shown in Supplementary Material, Appendix 3). A significantly higher proportion of episodes of SSTI required surgical intervention if associated with PVL^+^ MRSA compared to PVL^−^ MRSA (50% vs. 28%, *p* < 0.001).

**Figure 5.**
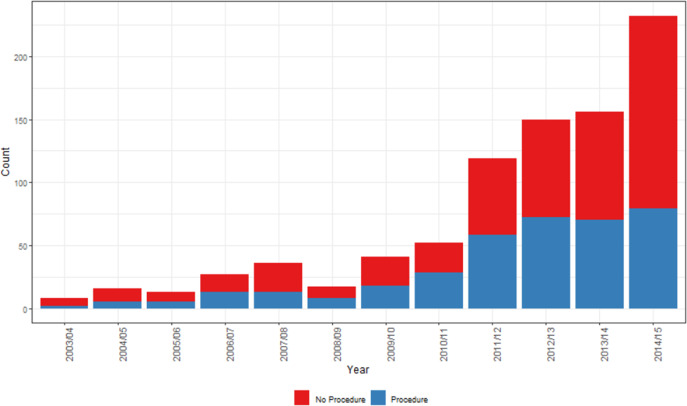
Annual count of CA-MRSA-related SSTI admissions requiring a procedure.

There was a notable plateau in the proportion of CA-MRSA-related SSTI cases requiring a procedure in 2014/2015. Despite a 49% increase in notifications between 2013/2014 and 2014/2015 (mostly attributable to PVL^+^ CA-MRSA), there was only a 13% increase in the number of SSTI-related procedures during the same period (Figure 5).

## Discussion

This study has linked Western Australian CA-MRSA notification data to inpatient and emergency department records to assess the use of health services due to, or related to, CA-MRSA infection over time. Our study has shown increasing CA-MRSA infections are associated with an increasing burden on hospitals. The rate of ED presentations and admissions designated as ‘CA-MRSA-related’ increased significantly over the study period in line with increasing CA-MRSA notifications. The increased incidence of CA-MRSA in the Kimberley region represents a continued burden on acute health services.

The most frequent principal reason for a CA-MRSA-related hospital admission was for SSTI, of which 43% annually required a procedure (range 25–53%). Similarly, CA-MRSA-related SSTIs represented the most frequent reason for presentation to an ED. This result is expected given the most CA-MRSA isolates from inpatients in a national survey were also isolated from SSTIs [24].

Our study quantifies the disproportionate burden of CA-MRSA infection in Aboriginal people living in the Kimberley region. Overall, CA-MRSA notification rates were significantly higher for Aboriginal people compared to non-Aboriginal people. Particularly between 2013/2014 and 2014/2015, there was a rapid increase in the notification rate of PVL^+^ CA-MRSA in the Aboriginal population, where approximately one in 13 of the Aboriginal population had a notification of PVL^+^ CA-MRSA at the end of the sub-study in 2014/2015. Aboriginal people are also over-represented in the proportion of individuals detected with CA-MRSA upon admission to the hospital or at presentation to the emergency department. Aboriginal people comprise approximately 42% of the region’s population [21] but 93% and 86% of total hospital admissions and emergency presentations among CA-MRSA cases, respectively.

Previous studies conducted in northern areas of Australia have reported an increasing incidence of CA-MRSA isolation from 2001 to 2011 in Aboriginal people [7] and a higher incidence of CA-MRSA infections compared to non-Aboriginal people [2]. A study conducted in the Northern Territory of Australia found high incidence rates of CA-MRSA infection in Aboriginal people were associated with measures of remoteness and socio-economic disadvantage [2]. Socio-behavioural factors such as overcrowding and poor sanitary conditions were likely to be more common in remote locations and may have perpetuated the ongoing transmission of circulating CA-MRSA and the prevalence of risk factors for CA-MRSA infection (e.g. scabies, streptococcal skin infection, ear infections) [25].

Our study demonstrates a shift in the molecular epidemiological profile of CA-MRSA notifications in the Kimberley region. From 2011 onwards, most isolates were PVL^+^. This predominance of PVL^+^ CA-MRSA was caused by the emergence of Queensland CA-MRSA [ST93-IV] and more recently, the WA 121 clone [ST5-IV]; rates of which increased 162- and 436-fold, respectively, over the sub-study period. In a 2012 survey, ST93-IV was identified as the most frequent clone circulating in Australia [10, 26].

Notably, however, the epidemiology of CA-MRSA and particularly ST93 differs in WA from other parts of Australia, with ST93-IV first identified in WA in 2003, several years after being identified in other Australian jurisdictions [12]. Although genetic data suggests the origins of ST93 were in WA, the clone was not detected by the state’s comprehensive surveillance systems until a decade later [12].

Overall, the study did not provide conclusive supporting evidence that PVL^+^ CA-MRSA causes more severe infections than PVL^−^ CA-MRSA. We have shown the increasing proportion of CA-MRSA notifications presenting to ED was similar for PVL^+^ and PVL^−^ notifications, and of those notifications who did present, PVL^−^ cases were more likely to be admitted. Emergency departments may be used as de facto primary care providers for residents; an increase in ED presentations is therefore not necessarily indicative of an increase in severe infections but rather just a marker of the increased notification rates within the region. Of those who presented to EDs, PVL^−^ notifications were more likely to be admitted to the hospital after controlling for age and Aboriginality.

There is no evidence of a proportional increase in hospital admissions due to PVL^+^ status, despite a notable increase in PVL^+^ notifications. The median LOS was the same (3 days) for PVL^−^ disease and PVL^+^ disease. While prima facie this suggests PVL^+^ CA-MRSA does not cause more severe SSTIs, data on associated procedures provide some contrasting evidence. A significantly higher proportion of PVL^+^ notifications required a procedure, suggesting PVL^+^ CA-MRSA infections may require more intensive management. The higher proportion of PVL^+^ MRSA SSTI patients who underwent procedures may be indicative of more severe skin infections; however, without the ability to review case notes and ascertain the reasoning behind the decision for surgical vs. medical management, the reasons for this observation remain unclear. As such, this apparent discrepancy in markers of severity may be due to surgical cases being discharged more rapidly after the procedure, whereas those being medically managed may require an extended stay until symptoms have attenuated.

Imported CA-MRSA clones comprised a small proportion of all CA-MRSA notifications in the Kimberley region, with a total of 19 notifications of the USA 300 [ST8-IV] clone, five of the Taiwan-A [ST-952-V_T_] clone, and six of the Bengal Bay [ST772-V] clone over the study period. This study was not able to determine the relative influence of disease incidence and greater awareness and testing frequency on the increasing number of CA-MRSA notifications in the Kimberley region; however, from the available literature and anecdotal evidence, it is likely that increased notification rates are due in part to a combination of more specimens being collected for microbiology investigation, but for the most part represent an increased prevalence in the community.

The frequent use of antimicrobial drugs to treat conditions such as scabies and other skin, respiratory, and ear infections may contribute to the emergence of new CA-MRSA clones through de novo resistance acquisition [2]. Two previously undetected PVL^+^ clones – Queensland CA-MRSA and WA 121 – emerged and proliferated throughout the surveillance period, with the first detections of each clone occurring in 2004/2005 and 2010/2011, respectively. The two clones continue to each represent approximately two-thirds of all notifications each (~70% of notifications in total) in 2022/2023. Clonal niches of CA-MRSA are well established, with distinct lineages emerging and proliferating within a discrete geographical area [27].

Our study has several limitations. The number of associated episodes of care presented in this study is considered an underestimate of the true burden of CA-MRSA infection on hospitals. We restricted the analysis to community-onset infections where a positive specimen was collected within 48 hours of admission [24] to reduce the influence of confounders such as age, co-morbid conditions, and hospital-acquired infections when comparing the characteristics of patients and episodes between clone PVL status. This method also increased our confidence that the reason for admission was caused by, or related to, the patient’s CA-MRSA infection and that the infection was acquired in the community rather than the hospital. As this study used secondary administrative data, it was not possible to account for unmeasured confounders, such as co-morbidities, that may have influenced previous contact with the hospital system, likelihood of being admitted to the hospital, and/or length of stay.

Also contributing to an underestimate of community and hospital burden, the use of notification data precludes the assessment of infections in individuals that did not present to health care or who were not swabbed, and this study was unable to include residents of the Kimberley who attend hospital care outside of WA. It was outside the scope of the study to examine the use of general practice services. In the Kimberley there are remote and town community clinics and several Royal Flying Doctor Service clinics that may treat patients for CA-MRSA, but these presentations were not included in this analysis.

## Conclusion

This study has shown increasing notifications of CA-MRSA in the Kimberley region of WA, with a particularly high burden for young Aboriginal people. A rapid increase in the number of notifications of PVL^+^ CA-MRSA raised concerns that the perceived increased virulence of PVL^+^ CA-MRSA would influence trends in hospital admissions, emergency presentations, and the severity of disease. This study found that increased notifications of CA-MRSA in the Kimberley region are associated with an increased burden on hospitals, particularly EDs.

There is no indication from our data that PVL^+^ CA-MRSA infections are associated with more invasive disease; however, there is evidence that these infections may be more likely to present as more severe SSTIs, requiring surgical intervention. Due to the difficulties associated with treatment of antimicrobial-resistant bacteria and the risk of transmission within the hospital environment, prevention of infection in the community is considered a more effective public health intervention. This study highlights the ongoing burden of increasing CA-MRSA infections on healthcare systems.

## Supporting information

Bloomfield et al. supplementary material Bloomfield et al. supplementary material

## Data Availability

Line-listed, linked data used in this manuscript are subject to Data Custodian approval for release per the WA *Health Services Act 2016.*

## Supplementary material

For supplementary material accompanying this paper visit http://doi.org/10.1017/S0950268824001201. click here to view supplementary material
