# Supplementary material for: Community-associated methicillin-resistant Staphylococcus aureus in the Kimberley region of Western Australia, epidemiology and burden on hospitals
Source: Epidemiol Infect. 2024 Nov 27;152:e147. doi: 10.1017/S0950268824001201 (PMC11626457; doi:10.1017/S0950268824001201)
Supplement: Bloomfield et al. supplementary material [file S0950268824001201sup001.docx]

# Appendices

**Appendix 1. Diagnoses of interest to CA-MRSA infection and classifying ICD-10-AM code(s) in the inpatient data collection**

| **Diagnosis of interest** | **ICD-10-AM code(s)** |
| --- | --- |
| Impetigo | L01 |
| Abscess, furuncle and carbuncle (“abscess”) | L02, J34.0, K61, K04.7, L05.0, N76.4, O91.1 |
| Cellulitis | L03, K12.2 |
| Arthritis, bursitis (“arthritis”) | M00.0, M00.9, M71.1 |
| Osteomyelitis | M86 |
| Sepsis | A41.0, A41.1, A41.2, A41.5, A41.9, O85 |
| Endocarditis | I33.0, I01.1 |
| Pneumonia | J15.2, J18.9 |
| Urinary tract infection | N39.0, O23.4 |
| Wounds | S01.0, S01.1, S01.41, S01.51, S01.88, S51.0, S51.88, S51.9, S61.0, S61.88, S61.9, S71.1, S81.0, S81.88, S91.0, S91.1, S91.3, S91.7, T01.3, T13.1, T79.3, T81.3, T81.41, T89.01 |

**Appendix 2. ICD-10-AM codes, skin and soft tissue infections**

| **ICD-10-AM Code** | **Description** |
| --- | --- |
| H60.0 | Abscess of external ear |
| J34.0 | Abscess, furuncle and carbuncle of nose |
| K12.2 | Cellulitis and abscess of mouth |
| K61.0 | Anal abscess |
| K61.1 | Rectal abscess |
| K61.2 | Anorectal abscess |
| K61.3 | Ischiorectal abscess |
| K61.4 | Intrasphincteric abscess |
| L00. | Staphylococcal scalded skin syndrome |
| L01.0 | Impetigo [any organism] [any site] |
| L01.1 | Impetiginisation of other dermatoses |
| L02.0 | Cutaneous abscess, furuncle and carbuncle of face |
| L02.1 | Cutaneous abscess, furuncle and carbuncle of neck |
| L02.2 | Cutaneous abscess, furuncle and carbuncle of trunk |
| L02.3 | Cutaneous abscess, furuncle and carbuncle of buttock |
| L02.4 | Cutaneous abscess, furuncle and carbuncle of limb |
| L02.8 | Cutaneous abscess, furuncle and carbuncle of other sites |
| L02.9 | Cutaneous abscess, furuncle and carbuncle, unspecified |
| L03.01 | Cellulitis of finger |
| L03.02 | Cellulitis of toe |
| L03.10 | Cellulitis of upper limb |
| L03.11 | Cellulitis of lower limb |
| L03.2 | Cellulitis of face |
| L03.3 | Cellulitis of trunk |
| L03.8 | Cellulitis of other sites |
| L03.9 | Cellulitis, unspecified |
| L08. | Other local infections of skin and subcutaneous tissue |
| L08.0 | Pyoderma |
| L08.1 | Erythrasma |
| L08.8 | Other specified local infections of skin and subcutaneous tissue |
| L08.9 | Local infection of skin and subcutaneous tissue, unspecified |
| L98.8 | Other specified disorders of skin and subcutaneous tissue |
| L98.9 | Disorder of skin and subcutaneous tissue, unspecified |
| L99. | Other disorders of skin and subcutaneous tissue in diseases classified elsewhere |
| L99.8 | Other specified disorders of skin and subcutaneous tissue in diseases classified elsewhere |
| T89.02 | Open wound with infection |

**Appendix 2. Procedures of interest to CA-MRSA infections and classifying ACHI code in the inpatient data collection**

| **ACHI code** | **Procedure description** | **Assigned category** |
| --- | --- | --- |
| 30216-01 | Aspiration of abscess of skin and subcutaneous tissue | Aspiration |
| 30216-02 | Other aspiration of skin and subcutaneous tissue | Aspiration |
| 50124-00 | Aspiration of joint or other synovial cavity, not elsewhere classified | Aspiration |
| 90725-00 | Aspiration of breast | Aspiration |
| 30023-00 | Excisional debridement of soft tissue | Incision/Excision/Drainage |
| 30023-01 | Excisional debridement of soft tissue involving bone or cartilage | Incision/Excision/Drainage |
| 30099-00 | Excision of sinus of skin and subcutaneous tissue | Incision/Excision/Drainage |
| 30103-00 | Excision of sinus involving soft tissue, not elsewhere classified | Incision/Excision/Drainage |
| 30223-01 | Incision and drainage of abscess of skin and subcutaneous tissue | Incision/Excision/Drainage |
| 30223-02 | Other incision and drainage of skin and subcutaneous tissue | Incision/Excision/Drainage |
| 30223-03 | Incision and drainage of abscess of soft tissue | Incision/Excision/Drainage |
| 30224-00 | Percutaneous drainage of abscess of soft tissue | Incision/Excision/Drainage |
| 31205-00 | Excision of lesion of skin and subcutaneous tissue of other site | Incision/Excision/Drainage |
| 31205-01 | Excision of ulcer of skin and subcutaneous tissue | Incision/Excision/Drainage |
| 31230-00 | Excision of lesion of skin and subcutaneous tissue of eyelid | Incision/Excision/Drainage |
| 31230-01 | Excision of lesion of skin and subcutaneous tissue of nose | Incision/Excision/Drainage |
| 31230-02 | Excision of lesion of skin and subcutaneous tissue of ear | Incision/Excision/Drainage |
| 31230-04 | Excision of lesion of skin and subcutaneous tissue of finger | Incision/Excision/Drainage |
| 31230-05 | Excision of lesion of skin and subcutaneous tissue of genitals | Incision/Excision/Drainage |
| 31235-00 | Excision of lesion of skin and subcutaneous tissue of other site of head | Incision/Excision/Drainage |
| 31235-01 | Excision of lesion of skin and subcutaneous tissue of neck | Incision/Excision/Drainage |
| 31235-02 | Excision of lesion of skin and subcutaneous tissue of hand | Incision/Excision/Drainage |
| 31235-03 | Excision of lesion of skin and subcutaneous tissue of leg | Incision/Excision/Drainage |
| 31235-04 | Excision of lesion of skin and subcutaneous tissue of foot | Incision/Excision/Drainage |
| 31350-00 | Excision of lesion of soft tissue, not elsewhere classified | Incision/Excision/Drainage |
| 31551-00 | Incision and drainage of breast | Incision/Excision/Drainage |
| 32174-01 | Drainage of perianal abscess | Incision/Excision/Drainage |
| 46519-00 | Incision and drainage of middle palmar, thenar or hypothenar spaces of hand | Incision/Excision/Drainage |
| 46525-00 | Incision and drainage of paronychia of hand | Incision/Excision/Drainage |
| 47918-00 | Radical excision of ingrown toenail bed | Incision/Excision/Drainage |
| 90545-00 | Incision of soft tissue of hand | Incision/Excision/Drainage |
| 90568-02 | Incision of soft tissue, not elsewhere classified | Incision/Excision/Drainage |
| 90575-00 | Excision of soft tissue, not elsewhere classified | Incision/Excision/Drainage |
| 90661-00 | Other incision of skin and subcutaneous tissue | Incision/Excision/Drainage |
| 90665-00 | Excisional debridement of skin and subcutaneous tissue | Incision/Excision/Drainage |
| 90686-01 | Non-excisional debridement of skin and subcutaneous tissue | Incision/Excision/Drainage |
| 96215-00 | Incision and drainage of lesion in oral cavity | Incision/Excision/Drainage |
| 97392-00 | Incision and drainage of abscess or cyst in oral cavity | Incision/Excision/Drainage |
